# Do health checks for adults with intellectual disability reduce emergency hospital admissions? Evaluation of a natural experiment

**DOI:** 10.1136/jech-2016-207557

**Published:** 2016-06-16

**Authors:** Iain M Carey, Fay J Hosking, Tess Harris, Stephen DeWilde, Carole Beighton, Sunil M Shah, Derek G Cook

**Affiliations:** Population Health Research Institute, St George's University of London, London, UK

**Keywords:** LEARNING DISABILITY, Health inequalities, Epidemiological methods

## Abstract

**Background:**

Annual health checks for adults with intellectual disability (ID) have been incentivised by National Health Service (NHS) England since 2009, but it is unclear what impact they have had on important health outcomes such as emergency hospitalisation.

**Methods:**

An evaluation of a ‘natural experiment’, incorporating practice and individual-level designs, to assess the effectiveness of health checks for adults with ID in reducing emergency hospital admissions using a large English primary care database. For practices, changes in admission rates for adults with ID between 2009–2010 and 2011–2012 were compared in 126 fully participating versus 68 non-participating practices. For individuals, changes in admission rates before and after the first health check for 7487 adults with ID were compared with 46 408 age-sex-practice matched controls. Incident rate ratios (IRRs) comparing changes in admission rates are presented for: all emergency, preventable emergency (for ambulatory care sensitive conditions (ACSCs)) and elective emergency.

**Results:**

Practices with high health check participation showed no change in emergency admission rate among patients with ID over time compared with non-participating practices (IRR=0.97, 95% CI 0.78 to 1.19), but emergency admissions for ACSCs did fall (IRR=0.74, 0.58 to 0.95). Among individuals with ID, health checks had no effect on overall emergency admissions compared with controls (IRR=0.96, 0.87 to 1.07), although there was a relative reduction in emergency admissions for ACSCs (IRR=0.82, 0.69 to 0.99). Elective admissions showed no change with health checks in either analysis.

**Conclusions:**

Annual health checks in primary care for adults with ID did not alter overall emergency admissions, but they appeared influential in reducing preventable emergency admissions.

## Introduction

Adults with intellectual disability (ID) experience high levels of morbidity, hospitalisation and premature mortality.^1^ In response to recommendations from the Disability Rights Commission,^2^ in 2009 the English National Health Service (NHS) introduced an annual health check scheme as a Directed Enhanced Service (DES) in primary care for adults with ID.^3^ This was intended to identify undetected health problems and improve prescribing and coordination with secondary care. Systematic reviews on the effectiveness of health checks in people with ID have confirmed that they are effective in identifying new health problems, improving uptake of preventive interventions and improving indicators of process of care.^4^ However, there is little evidence on their effectiveness in modifying outcomes such as hospitalisation,^5^ which is important for patients, carers and the health services. With only half of the eligible adults receiving health checks by 2011–2012,^6^ this provided an opportunity to evaluate the scheme by viewing it as a ‘natural experiment’.

In this paper, we use a robust observational methodology, using practice-level and individual-level designs, to assess whether the introduction of health checks in 2009 reduced emergency hospitalisation for adults with ID. We first compare high with low uptake practices, evaluating change in admission rates for all adults with ID, controlling for underlying differences between practices. However, the possibility remains that participating practices improved the care of their patients with ID independent of introducing health checks. Therefore, we also present a matched cohort study comparing change in admission rates of individuals with ID who had health checks to that seen for a matched group of patients without ID, controlling for secular trends in practice care or hospital admissions. Finally, a second matched cohort study for individuals with ID not receiving health checks is then used to confirm the specificity of findings to those having a health check only.

## Methods

### Data source

The Clinical Practice Research Datalink (CPRD) is a large primary care database representative of the UK population.^7^ We included 343 practices in England recording data on 1 January 2009, anonymously linked to Hospital Episodes Statistics (HES) data. HES records clinical and administrative information on all NHS-funded inpatient episodes, and allows for identification on method of admission (eg, emergency), in addition to the primary reason for the admission.

### Identification of patients with ID and their health checks

We have previously detailed our methodology for identifying adults (aged 18–84) with ID in CPRD in England.^8^ Briefly, we included all codes used by the Quality and Outcome Framework (QOF) for learning disability,^9^ plus additional codes for conditions usually associated with ID such as chromosomal and metabolic disorders (see online supplementary e-table 1). Health checks were identified by specific Read codes used by practices to facilitate future payment. We only included health checks from 1 April 2009, the point from when practices received remuneration for carrying them out.

10.1136/jech-2016-207557.supp1 Supplementary tables

We classified patients with ID with high levels of support needs based on either a record of severe or profound ID or, where no record of severity was available (59%), at least two of the following: cerebral palsy/significant mobility problem, severe visual impairment, severe hearing impairment, epilepsy (excluding absence seizures), a continence problem, or use of percutaneous endoscopic gastrostomy feeding (see online supplementary e-table 2). Patients with ID were estimated to be living in a communal setting by specific Read codes (see online supplementary e-table 3), or the presence of three or more people with ID with the same address flag.

### Hospital admission outcomes

Our main outcome was a count of emergency hospital admissions, defined as distinct periods of care on the HES record. We were also interested in emergency admissions for ambulatory care sensitive conditions (ACSCs),^10^ which are thought to be potentially preventable with better clinical management. We included 20 widely used ACSCs, adding three further conditions (constipation, aspiration, gastro-oesophageal reflux disease) which are more relevant reasons for admission among adults with ID.^11^ We identified these using the primary International Classification of Diseases (ICD)-10 diagnosis for the first episode of the hospitalisation (see online supplementary e-table 4). We also analysed elective admissions as an outcome, to test whether health checks had an impact on this aspect of care.

### Practice-level assessment of health checks

**Figure 1:**
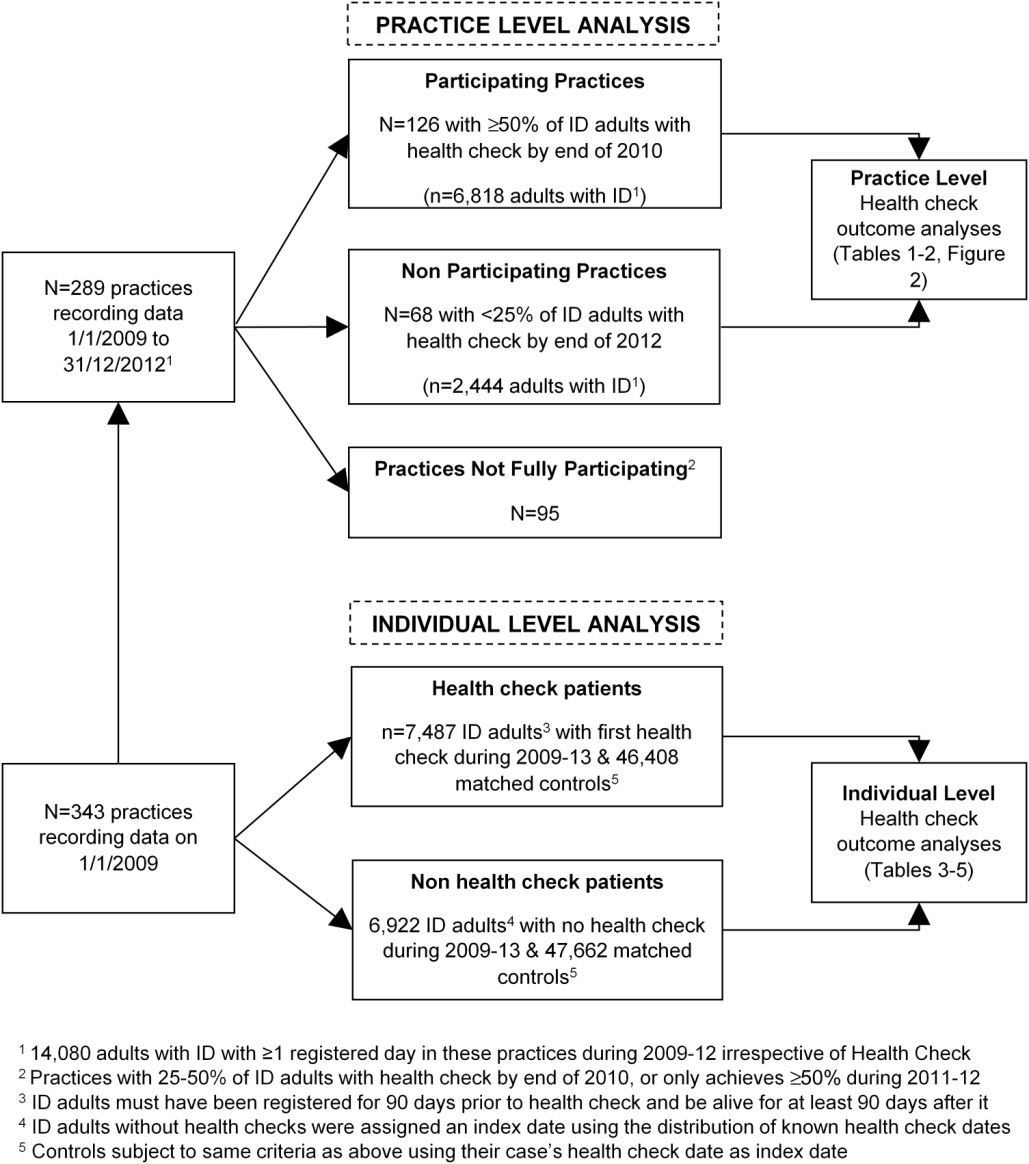
Summary of number of practices, adults with intellectual disability (ID) and matched controls used in analyses.

We classified practice participation in the DES by calculating the percentage of patients registered on 1 January 2009 on the QOF learning disability register that subsequently received a health check. For this analysis, we restricted to 289 practices with complete data from 1 January 2009 to 31 December 2012, including all adults with IDs irrespective of whether they received a health check (figure 1). We defined full practice participation (n=126) as ≥50% of their adults with ID having a health check by 31 December 2010. Practices (n=68) with <25% adults having a health check by 31 December 2012 were classed as non-participating, with the remainder (n=95) having participation rates of 25–50%. We then compared practice hospital admission rates (total admissions divided by total registration time) in 2011–2012 vs 2009–2010 between practices fully participating and non-participating.

### Individual analysis of first health check

For our analysis of individuals, we carried out a matched cohort analysis that compared within participant, the rate of admission after the first recorded health check from 1 January 2009 to 31 December 2013, with that seen before the health check (figure 1). Up to seven controls (with no record of ID) were matched on age, sex and practice to control for any temporal trends in admissions during the study. In total, 7487 adults with ID aged 18–84 with a first health check were identified and matched to 46 408 controls. We excluded the period 30 days on either side of the health check to avoid it directly leading to an admission, or being the result of a recent discharge from hospital. All patients were required to be registered for at least 90 days prior to the health check, and be alive for 90 days after it. All patients were followed to 31 December 2013, or their death if it was earlier. Those who de-registered from their practice were still included in the follow-up as linkage to hospital admissions continues as long they remain resident in England.

Finally, we carried out a complementary analysis using 6922 adults with ID without health checks (figure 1). We allocated a random index date based on the known dates of the health checks, and similarly matched them to 47 662 population controls. We then repeated the above analysis using the non-health check adults with ID and their controls to check whether any observed changes in admissions for adults with ID were specific to those receiving health checks only.

### Statistical analysis

The analyses used a conditional Poisson model (xtpoisson, Stata V.13) to compare the rate of change over time at a practice or individual level. At the practice level, these were conditioned on practice, and all admissions from patients with ID were counted, using an offset term to account for total time registered. The effect of practice participation on hospital admissions was estimated by the interaction between practice participation (fully vs none) and period (2011–2012 vs 2009–2010). At the individual level, we conditioned on individual as opposed to matchset, as accounting for the matching variables is not paramount in matched cohort analyses.^12^ This model was fitted to adults with ID and controls separately, estimating the individual change in hospital admission rate after as compared with before the health check, with an offset accounting for time registered. A combined model of adults with ID and controls with a case-period interaction provides an estimate of the effect of health checks on admission rates among adults with ID, adjusted for temporal trends in admissions. All models used a sandwich estimator to obtain robust standard errors.

## Results

### Practice-level analyses of health checks and hospital admissions

Practices fully participating in health checks compared with those not participating (table 1) were more likely to have larger numbers of adults with ID in their practice, as well as higher percentages recorded of those living in communal establishments (median 15.8% vs 5.9%) and having high levels of support need (median 22.2% vs 15.2%).

**Table 1:** Summary of adults with ID in each practice by practice-level participation in health checks

	All practices (N=289)		Non-participating practices (N=68)		Partial participating practices (N=95)		Fully participating practices (N=126)	
	Median	IQR	Median	IQR	Median	IQR	Median	IQR
Adults with ID summarised at practice level*								
Total registered during 2009–2012†	43.0	25.0–64.0	36.0	16.0–50.0	46.0	31.0–64.0	45.0	24.0–79.0
Number registered on 1 January 2009 only	34.0	19.0–52.0	26.5	12.5–39.5	34.0	23.0–53.0	38.0	19.0–61.0
Per cent with health check by end of 2010	43.1	1.6–65.8	0.0	0.0–0.0	22.2	4.3–41.7	69.5	60.0–80.0
Per cent with health check by end of 2012	66.7	28.6–81.8	0.0	0.0–11.8	58.6	41.0–68.8	81.8	74.2–87.9
Mean age	41.6	38.7–44.8	41.9	38.9–45.8	40.5	37.5–43.8	42.6	39.4–45.0
Per cent of male	57.6	50.0–64.3	55.6	50.0–64.5	58.3	50.0–63.2	57.5	50.0–65.0
Per cent of high levels of support needs	18.8	10.5–27.0	15.2	8.2–21.6	17.4	10.2–27.8	22.2	14.0–30.0
Per cent of communal establishment residence	9.7	0.0–26.4	5.9	0.0–23.1	8.6	0.0–21.4	15.8	2.3–34.2

Fully participating practices had ≥50% of their adults with ID with a health check by end of 2010. Non-participating practices had <25% of their adults with ID with a health check by end of 2012. 95 (partial participating) practices did not meet either criterion. In all 72 of the 289 practices had zero participation by 2010, compared to 35 by 2012.

*Medians are calculated among all adults with ID registered on 1 January 2009, except for the ‘number registered during 2009–2012’. First, a mean is calculated at practice level, and then a median of the practice means is calculated.

†Patients who spent at least 1 day registered during 2009–2012.

ID, intellectual disabillity.

**Figure 2:**
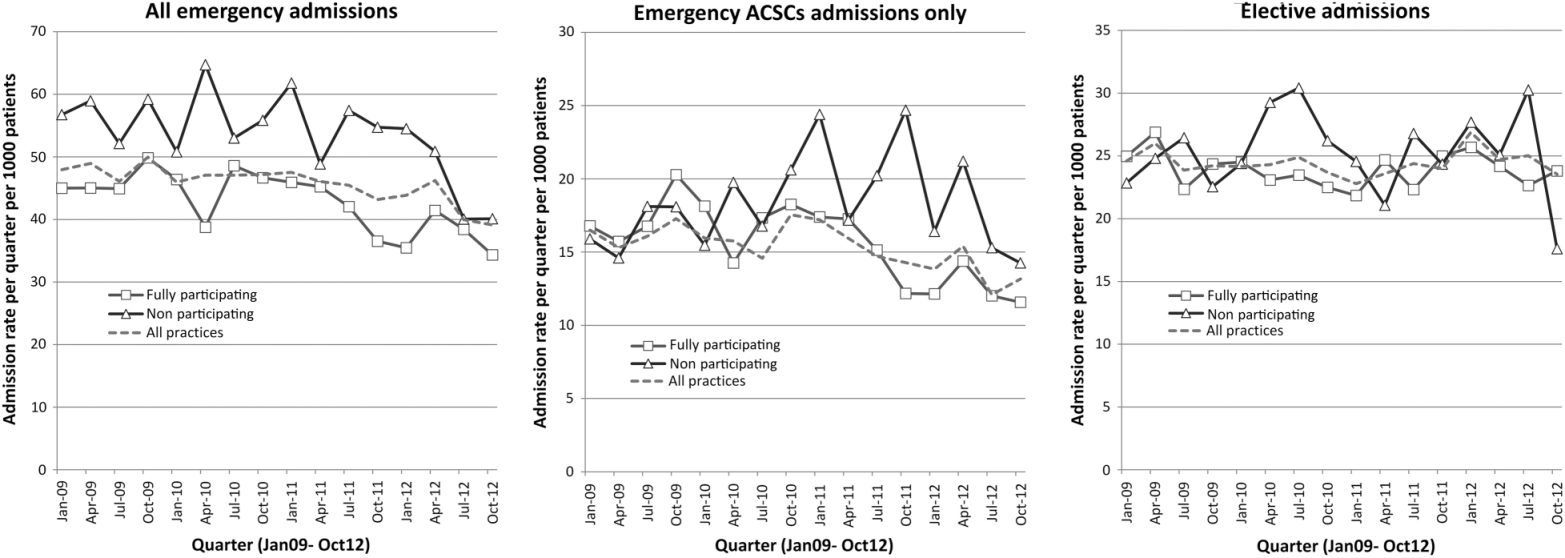
Hospital admissions (all emergency, emergency for ACSCs (ambulatory care sensitive conditions), elective) in each quarter during 2009–2012 by practice level of participation in health checks.

A summary of hospital admissions (all emergency, emergency ACSCs, elective) among adults with ID during 2009–2012 is shown in figure 2 and analysed in table 2. Emergency admission rates calculated in each quarter (figure 2) tended to fall over time. This is summarised annually in table 2 as a fall from 191.1 per 1000 patients per year in 2009–2010, to 176.7 in 2011–2012. Non-participating health check practices had consistently higher emergency admission rates throughout than those fully participating (figure 2), with both groups experiencing a similar fall over time (incident rate ratio (IRR)=0.97, 95% CI 0.78 to 1.19).

**Table 2:** Hospital admissions in 2011–2012 vs 2009–2010 by practice level of participation in health checks

Practice status	Outcome	Annual rate in 2009–2010 per 1000 person years	Annual rate in 2011–2012 per 1000 person years	Practice period IRR* (95% CI)	Practice change in IRR (95% CI) for fully participating vs non-participating practices†
All practices (N=289)	All emergency admissions	191.1	176.7	0.92 (0.86 to 0.99)	–
	Emergency ACSCs only‡	64.9	58.6	0.91 (0.82 to 1.00)	–
	All elective admissions§	117.1	119.2	1.02 (0.95 to 1.09)	–
Fully participating practices (N=126)	All emergency admissions	183.6	160.6	0.88 (0.80 to 0.96)	0.97 (0.78 to 1.19)
	Emergency ACSCs only‡	69.2	56.3	0.82 (0.72 to 0.92)	0.74 (0.58 to 0.95)
	All elective admissions§	112.4	114.0	1.02 (0.92 to 1.14)	1.02 (0.84 to 1.25)
Non-participating practices (N=68)	All emergency admissions	226.9	205.3	0.90 (0.75 to 1.09)	1.00
	Emergency ACSCs only‡	70.1	77.1	1.10 (0.89 to 1.36)	1.00
	All elective admissions§	125.9	127.3	1.00 (0.85 to 1.19)	1.00

Fully participating practices had >50% of their patients with ID with a health check by end of 2010. Non-participating practices had <25% of their patients with ID with a health check by end of 2012. Ninety-five practices did not meet either criteria and were excluded from the comparison.

*This represents the within-practice change in admission post-health check compared with prehealth check estimated from the conditional Poisson model.

†This represents the within-practice post-health check change in admissions between the fully participating practices versus the non-participating practices estimated from the conditional Poisson model.

‡For definition of ambulatory care sensitive conditions, please refer to e-table 1.

§Exclude patients with abnormally high elective rates (average >6/year).

ACSC, ambulatory care sensitive condition; ID, intellectual disability; IRR, incident rate ratio.

When emergency admissions for ACSCs were considered the pattern was different (figure 2 and table 2). While these admissions had fallen among those fully participating in health checks (69.2 in 2009–2010 to 56.3 in 2011–2012 per 1000 patients), they tended to rise in practices not participating (70.1 to 77.1 per 1000 patients). A statistical comparison of the difference in this change showed an overall benefit of greater practice participation (IRR=0.74, 95% CI 0.58 to 0.95). There was no evidence of any difference in the change over time in elective admissions between fully participating and non-participating practices (IRR=1.02, 95% CI 0.84 to 1.25).

### Characteristics of adults with ID with and without health checks

Among the 7487 adults with ID with a first health check between 1 April 2009 and 31 March 2013, the average age was 42.6 years (SD=15.4), with 57.5% being male (table 3). Almost 3 in 10 were classified as having high levels of support needs, with a similar proportion identified as being resident in a communal establishment. By contrast, the 6922 ID adults without a health check were younger (mean=39.0) and less likely to have high levels of support needs or communal living recorded on their record.

**Table 3:** Characteristics of registered adult patients with ID by whether they had a health check between April 2009 and March 2013

	Patients with with ID with a health check		Patients with ID without a health check	
Individual characteristic	n	Per cent	N	Per cent
All	7487	100	6922	100
Gender				
Women	3183	42.5	2889	41.7
Men	4304	57.5	4033	58.3
Age at health check/index date (years)				
18–34	2579	34.5	3159	45.6
35–54	3136	41.9	2432	35.1
55–84	1772	23.7	1331	19.2
Down's syndrome				
No	6573	87.8	6283	90.8
Yes	914	12.2	639	9.2
Autism spectrum disorder				
No	6744	90.1	6423	92.8
Yes	743	9.9	499	7.2
High support needs*				
No	5452	72.8	6031	87.1
Yes	2035	27.2	891	12.9
Lives in communal establishment				
Not recorded	5574	74.5	6111	88.3
Yes	1913	25.6	811	11.7

*Has been classed as having severe or profound ID by the GP or has two or more of the following in addition to an ID diagnosis: epilepsy, cerebral palsy or significant mobility problem (wheelchair use or greater problem), severe visual impairment, severe hearing impairment, a continence problem, or use of PEG feeding.

ID, intellectual disability; GP, general practitioner; PEG, percutaneous endoscopic gastrostomy.

### Individual analyses of health checks and hospital admissions

Hospital admission rates before and after the health check are summarised in table 4, and also for adults without health checks using their random index date. For adults with a health check, all emergency admissions rose by 22% from 145.7 to 173.2 annually per 1000 patients. By contrast, in their matched controls the rate increased by 27% from 58.9 to 70.2 (data not shown). Therefore, in the combined Poisson model, the interaction for the impact of health checks on adults with ID is estimated to be under 1 (IRR=0.96, 95% CI 0.87 to 1.07). Adults with ID without health checks had higher overall admission rates for emergency admission (eg. 186.0 vs 145.7 pre index date), and a slight increase in admission rate post-index date relative to their controls (IRR=1.05, 95% CI 0.94 to 1.17).

**Table 4:** Summary of hospital admission rates in adults with ID pre-health check and post-health check, or index date for those without a health check

		Pre-health check		Post-health check			Change in IRR (95% CI) vs age-sex-practice matched controls†
	Outcome	Total admissions	Annual rate/1000	Total admissions	Annual rate/1000	Period IRR* (95% CI)	
Patients with ID with health check (n=7487)	All emergency admissions	1673	145.7	3840	173.2	1.22 (1.11 to 1.34)	0.96 (0.87 to 1.07)
	Emergency ACSCs only‡	602	52.4	1314	59.3	1.11 (0.95 to 1.29)	0.82 (0.69 to 0.99)
	All elective admissions§	1328	115.9	2703	122.4	1.11 (1.01 to 1.21)	0.96 (0.87 to 1.06)
Patients with ID without health check but assigned a random index date (n=6922)	All emergency admissions	1836	186.0	4263	212.2	1.20 (1.09 to 1.32)	1.05 (0.94 to 1.17)
	Emergency ACSCs only‡	520	52.7	1340	66.7	1.35 (1.14 to 1.60)	1.11 (0.92 to 1.36)
	All elective admissions§	1170	119.1	2567	128.4	1.02 (0.93 to 1.12)	0.90 (0.81 to 1.00)

Mean follow-up time was—patients with ID with a health check: 560 days (pre), 1081 (post). Patients with ID without a health check: 521 days (pre), 1059 (post).

*This represents the within-person change in admission post-health check compared with pre-health check estimated from the conditional Poisson model.

†This represents the within-person post-health check change in admissions between the patients with ID and their respective controls (n=46 408 for patients with ID with health check, n=47 622 for patients with ID without health check) estimated from the conditional Poisson model.

‡For definition of ambulatory care sensitive conditions please refer to e-table 4.

§Excludes patients with abnormally high elective rates (average >6/year).

ACSC, ambulatory care sensitive condition; ID, intellectual disability; IRR, incident rate ratio.

Emergency admissions for ACSCs among adults with health checks showed an association with change in admission rate posthealth check compared with controls (IRR=0.82, 95% CI 0.69 to 0.99). This trend was not replicated in adults with ID without a health check (IRR=1.11, 95% CI 0.92 to 1.36). The change in elective admission rate was similar between ID adults with health checks and controls (IRR=0.96, 95% CI 0.87 to 1.06).

Table 5 summarises the estimate of the impact of health checks on emergency hospital admissions stratified by individual characteristics, both for adults with ID with and without health checks. A significant rise in admissions among Down's syndrome adults with health checks compared with their controls (IRR=1.55, 95% CI 1.15 to 2.08), was replicated among Down's adults without health checks (IRR=1.55), suggesting a trend specific to this group. By contrast, while health checks reduced emergency admissions among adults with ID with high levels of support needs (IRR=0.80, 95% CI 0.67 to 0.95), this was not replicated in similarly defined patients with ID without health checks (IRR=1.07, 95% CI 0.85 to 1.35). A further analysis of patients with ID with high levels of support needs receiving health checks also suggested a decrease in their emergency admissions for ACSCs compared with controls (IRR=0.76, 95% CI 0.56 to 1.01, data not shown).

**Table 5:** Interaction IRRs comparing the change in emergency hospital admission rates post-health check between adults with ID and matched controls, stratified by individual characteristics

	Patients with ID with a health check	Patients with ID without a health check
Status at time of health check	Change in IRR (95% CI) vs age-sex-practice matched controls*	Change in IRR (95% CI) vs age-sex-practice matched controls*
Gender		
Women	1.07 (0.92 to 1.25)	1.13 (0.95 to 1.34)
Men	0.88 (0.76 to 1.01)	0.98 (0.85 to 1.13)
Age (years)		
18–34	1.01 (0.81 to 1.25)	0.97 (0.80 to 1.16)
35–54	0.95 (0.80 to 1.13)	1.12 (0.92 to 1.34)
55–84	0.96 (0.81 to 1.14)	0.96 (0.78 to 1.18)
Down’s syndrome		
No	0.91 (0.82 to 1.02)	1.01 (0.90 to 1.14)
Yes	1.55 (1.15 to 2.08)	1.55 (1.08 to 2.22)
Autism spectrum disorder		
No	0.95 (0.85 to 1.05)	1.04 (0.93 to 1.16)
Yes	1.18 (0.76 to 1.82)	1.25 (0.75 to 2.08)
High support needs†		
No	1.06 (0.93 to 1.22)	1.03 (0.90 to 1.17)
Yes	0.80 (0.67 to 0.95)	1.07 (0.85 to 1.35)
Lives in communal establishment		
Not recorded	0.91 (0.80 to 1.03)	1.02 (0.90 to 1.15)
Yes	1.13 (0.92 to 1.38)	1.22 (0.92 to 1.62)

*This represents the within-person post-health check change in admissions between the patients with ID and their respective controls (n=46 408 for patients with ID with health check, n=47 622 for patients with ID without health check) estimated from the conditional Poisson model.

†Has been classed as having severe or profound ID by the GP or has two or more of the following in addition to an ID diagnosis: epilepsy, cerebral palsy or significant mobility problem (wheelchair use or greater problem), severe visual impairment, severe hearing impairment, a continence problem, or use of PEG feeding.

ID, intellectual disability; IRR, incidence rate ratio; GP, general practitioner; PEG, percutaneous endoscopic gastrostomy.

## Discussion

In this study, we found little evidence to suggest that the introduction of incentivised health checks by NHS England for adults with ID in 2009 had any discernible impact on subsequent overall emergency or elective admissions. However, when we only considered potentially preventable emergency admissions (ACSCs) we found that practices which were fully participating in health checks experienced a greater fall in admissions than those not participating. This beneficial association with preventable admissions was replicated when we looked directly at individuals with ID who had a recorded health check. This analysis also suggested a wider benefit of health checks on all emergency admissions among those with more complex health needs.

We believe our study is the first to report benefits of health checks for adults with ID on a health outcome as opposed to process measures.^13^ While a systematic review has shown the effectiveness of health checks in detecting unrecognised health needs in people with ID,^4^ it highlighted the lack of evidence regarding whether their provision translated into important longer term benefits, such as a reduction in avoidable hospitalisations or mortality. The evidence for effectiveness of health checks in general adult populations is similarly uncertain, with no evidence that they reduce mortality, hospitalisation or disability.^14^ In the UK, NHS health checks for 40–74 years old have been shown to increase the identification of cardiovascular risk factors in a large untreated population,^15^ but their impact of longer term outcomes is still unclear.

Reducing emergency hospital admissions is a major international concern to contain healthcare costs, but evidence for successful community interventions is limited.^16^ While our primary outcome of emergency hospital admission showed no change after introduction of health checks for participants with ID, evidence for a reduction in potentially preventable admissions was consistent in all our analyses and plausible. Given that admissions for ACSCs represent about 1 in 5 emergency admissions in the UK,^10^ it is unsurprising that we did not detect a change for the broader group. Among adults with ID in our study, admissions for epilepsy contributed about 45% of emergency admissions for ACSCs, so one possible explanation is that health checks are facilitating better overall management of epilepsy and seizures among patients with ID. This would be an important benefit, as improved service provision of patients with ID with epilepsy has been identified as a mechanism for reducing excess mortality among all people with ID.^5^

Our study reached a similar conclusion from two different analytic strategies, one based on practice comparisons and the other on individuals. As these used slightly different patient groups and definitions of time, this outcome would not necessarily be expected. For example, individual analyses suggested emergency hospital admissions were rising among patients with ID post-health check, while practice-level analyses showed a fall during 2011–2012. The rise in admissions in the same individuals is partly explained by their ageing over time, plus the requirement to be alive at the health check, resulting in deaths only post-health check (and associated admissions). By contrast, practice trends were based on a fluid group of all patients with ID aged 18–84 years in each year, keeping average age effectively constant and allowing deaths within the year.

Our study has some limitations. We were not able to comment on the quality or overall content of the health checks that have taken place. Although there is published guidance on what the general practitioner (GP) should cover during a health check,^3^ a general observation from our data extract is that there is substantial variation in what is recorded, which is likely to mirror what is taking place in the health checks. We have not attempted to make any economic costing of the effectiveness of the health check scheme. A small Scottish trial of nurse delivered health checks for adults with ID demonstrated cost-effectiveness compared with standard care.^17^ However, they did not include hospitalisation costs, except accident and emergency attendances, which may have led to them underestimating potential economic savings.

The analysis at practice level was unmatched, and likely subject to residual confounding from unmeasured factors at both practice and individual level, as we would expect practices that participate in the DES to be different than those that do not, and possibly have differing characteristics of patients with ID. For example, practices that went on to regularly carry out health checks in our study already had lower emergency hospital admissions rates among their patients with ID at the outset in 2009. These practices might have further reduced admissions anyway, and subsequent adoption of health checks may simply be a marker of other improvements in their care over the study period.

In order to control for any practice level-changes over time, we matched individuals with ID receiving health checks to population controls in the same practice. This analysis now adjusts for any change (artefact or real) across practice or hospitals during the study. However, it fails to account for changes specific to people with ID that may have happened in the UK in light of several high profile reports during this period.^2^ ^18^ Therefore, we similarly analysed patients with ID without health checks, assigning them a random date instead of a health check date. Since this group showed no fall in ACS admissions compared with their controls, it provided additional evidence for the effectiveness of health checks. This contrasts with our finding that adults with Down's syndrome increased emergency admissions by 55% post-health check, but since a similar increase was seen in Down's adults *without* health checks, we concluded the trend was specific to Down's and not health checks. This increase may reflect the premature ageing associated with Down's such as early onset Alzheimer's disease,^19^ combined with better survival into middle age in part due to advances in childhood cardiac surgery.^20^

In England, an increasing number of adults with ID now live in the community, and as a result, the GP's role in managing their health has increased. Preliminary work around the time of the introduction of health checks in 2009 in England suggested there were no associated higher costs in terms of service use,^21^ however costs implications since are not clear and should be evaluated. It has been argued that regular health checks for adults with ID are an efficient way of closing the health inequality gap that this group may experience; however, this may also be widened if more easily managed patients are more likely to get health checks.^22^ In our study, the decrease in emergency admission rates for ACSCs was more marked (26%) when we directly compared participating with non-participating practices, which suggests that there may be a ‘practice-level benefit’ of health checks, where changes in care have benefited all patients with ID within the practice irrespective of whether they have the health check. However, this may be an oversimplification, as a recent serious case review in the UK into the deaths of two adults with ID found that they had been invited to a health check but had failed to attend.^23^ Interestingly, our analysis of individuals suggested that health checks produced the greatest benefit in reducing emergency admission to hospital in those with more severe and complex needs.

In summary, to continue to successfully address issues of health inequality and discrimination for adults with ID, the policy implications from our results are: (1) to increase the practice uptake of the health check DES from current levels (<60%) towards a suggested and necessary target of 90%;^22^ (2) to ensure that all eligible adults, especially those with the most severe or complex needs, receive an annual health check within practices who participate in the DES.

**What is already known on this subject**

- A systematic review of the impact of health checks for people with intellectual disabilities (IDs) in 2014 concluded that while health checks were ‘effective in identifying previously unrecognised health needs, including life-threatening conditions’, very few studies had ‘evaluated the extent to which providing health checks for people with IDs leads to health benefits either in the short or long term’. We were not aware of any study that used emergency hospitalisations as an outcome when evaluating health checks.

**What this study adds**

- While there was no evidence to suggest that health checks had an impact on overall emergency hospital admissions for adults with ID, the study did reveal a reduction in preventable emergency hospitalisations after the introduction of health checks. These findings should encourage further implementation of health checks to include all general practices in England, in addition to wider participation within practices already carrying them out.
