# Supplementary material for: Do health checks for adults with intellectual disability reduce emergency hospital admissions? Evaluation of a natural experiment
Source: J Epidemiol Community Health. 2016 Jun 16;71(1):52–8. doi: 10.1136/jech-2016-207557 (PMC5256310; doi:10.1136/jech-2016-207557)
Supplement: Supplementary tables [file jech-2016-207557supp_tables.pdf]

E-Table 1: Read codes used to define intellectual disability

Read Code	Description	Used by QOF's Learning Disability*
**13Z3.00**	Low I.Q.	
**6664.00**	Mental handicap problem	
**69DB.00**	Learnin disability health exam	Health Check
**918e.00**	On learning disability register	Register
**9HB..00**	Learning disabilities administration status	
**9HB0.00**	Learning disabilities health action plan declined	
**9HB1.00**	Learning disabilities health action plan offered	
**9HB2.00**	Learning disabilities health action plan reviewed	
**9HB3.00**	Learning disabilities health assessment	Health Check
**9HB4.00**	Learning disabilities health action plan completed	
**9HB5.00**	Learning disabilities annual health assessment	Health Check
**9HB6.00**	Learning disabilities annual health assessment declined	
**9HB6.11**	Learning disabilities annual health check declined	
**9HB7.00**	Did not attend learning disabilities annual health assessmnt	
**9HB7.11**	Did not attend learning disabilities annual health check	
**9hL..00**	Exception reporting: learning disability quality indicators	
**9hL0.00**	Exc learn disability quality indicators: informed dissent	
**9hL1.00**	Exc learn disability quality indicators: patient unsuitable	
**9mA..00**	Learning disability annual health check invitation	
**9mA0.00**	Learning disability annual health check verbal invitation	
**9mA1.00**	Learning disability annual health check telephone invitation	
**9mA2.00**	Learning disability annual health check letter invitation	
**9mA2000**	Learning disability annual health check invtation 1st letter	
**9mA2100**	Learning disability annual health check invtation 2nd letter	
**9mA2200**	Learning disability annual health check invtation 3rd letter	
**C03..11**	Cretinism	
**C031.00**	Goitrous cretin	
**C03z.12**	Cretinism	
**C372.11**	Lesch - Nyhan syndrome	
**C372000**	Hypoxanthine-guanine-phosphoribosyltransferase deficiency	
**C372011**	Lesch - Nyhan syndrome	
**C372300**	Lesch-Nyhan syndrome	
**C372z00**	Other disorder of purine or pyrimidine metabolism NOS	
**E141.00**	Disintegrative psychosis	
**E141.11**	Heller's syndrome	
**E141000**	Active disintegrative psychoses	
**E141100**	Residual disintegrative psychoses	
**E141z00**	Disintegrative psychosis NOS	
**E3...00**	Mental retardation	Register
**E30..00**	Mild mental retardation, IQ in range 50-70	Register
**E30..11**	Educationally subnormal	Register
**E30..12**	Feeble-minded	Register
**E30..13**	Moron	Register
**E31..00**	Other specified mental retardation	Register

Read Code	Description	Used by QOF's Learning Disability*
**E310.00**	Moderate mental retardation, IQ in range 35-49	Register
**E310.11**	Imbecile	Register
**E311.00**	Severe mental retardation, IQ in range 20-34	Register
**E312.00**	Profound mental retardation with IQ less than 20	Register
**E312.11**	Idiocy	Register
**E31z.00**	Other specified mental retardation NOS	Register
**E3y..00**	Other specified mental retardation	Register
**E3z..00**	Mental retardation NOS	Register
**Eu7..00**	[X]Mental retardation	Register
**Eu70.00**	[X]Mild mental retardation	Register
**Eu70.11**	[X]Feeble-mindedness	Register
**Eu70.12**	[X]Mild mental subnormality	Register
**Eu70000**	[X]Mld mental retard with statement no or min impairm behav	Register
**Eu70100**	[X]Mld mental retard sig impairment behav req attent/treatmt	Register
**Eu70y00**	[X]Mild mental retardation, other impairments of behaviour	Register
**Eu70z00**	[X]Mild mental retardation without mention impairment behav	Register
**Eu71.00**	[X]Moderate mental retardation	Register
**Eu71.11**	[X]Moderate mental subnormality	Register
**Eu71000**	[X]Mod mental retard with statement no or min impairm behav	Register
**Eu71100**	[X]Mod mental retard sig impairment behav req attent/treatmt	Register
**Eu71y00**	[X]Mod retard oth behav impair	Register
**Eu71z00**	[X]Mod mental retardation without mention impairment behav	Register
**Eu72.00**	[X]Severe mental retardation	Register
**Eu72.11**	[X]Severe mental subnormality	Register
**Eu72000**	[X]Sev mental retard with statement no or min impairm behav	Register
**Eu72100**	[X]Sev mental retard sig impairment behav req attent/treatmt	Register
**Eu72y00**	[X]Severe mental retardation, other impairments of behaviour	Register
**Eu72z00**	[X]Sev mental retardation without mention impairment behav	Register
**Eu73.00**	[X]Profound mental retardation	Register
**Eu73.11**	[X]Profound mental subnormality	Register
**Eu73000**	[X]Profound ment retrd wth statement no or min impairm behav	Register
**Eu73100**	[X]Profound ment retard sig impairmnt behav req attent/treat	Register
**Eu73y00**	[X]Profound mental retardation, other impairments of behavr	Register
**Eu73z00**	[X]Prfnd mental retardation without mention impairment behav	Register
**Eu7y.00**	[X]Other mental retardation	Register
**Eu7y000**	[X]Oth mental retard with statement no or min impairm behav	Register
**Eu7y100**	[X]Oth mental retard sig impairment behav req attent/treatmt	Register
**Eu7yy00**	[X]Other mental retardation, other impairments of behaviour	Register
**Eu7yz00**	[X]Other mental retardation without mention impairment behav	Register
**Eu7z.00**	[X]Unspecified mental retardation	Register
**Eu7z.11**	[X]Mental deficiency NOS	Register
**Eu7z.12**	[X]Mental subnormality NOS	Register
**Eu7z000**	[X]Unsp mental retard with statement no or min impairm behav	Register
**Eu7z100**	[X]Unsp mentl retard sig impairment behav req attent/treatmt	Register
**Eu7zy00**	[X]Unspecified mental retardatn, other impairments of behav	Register

Read Code	Description	Used by QOF's Learning Disability*
**Eu7zz00**	[X]Unsp mental retardation without mention impairment behav	Register
**Eu81400**	[X]Moderate learning disability	Register
**Eu81500**	[X]Severe learning disability	Register
**Eu81600**	[X]Mild learning disability	Register
**Eu81700**	[X]Profound learning disability	Register
**Eu81z00**	[X]Developmental disorder of scholastic skills, unspecified	Register
**Eu81z11**	[X]Learning disability NOS	Register
**Eu81z12**	[X]Learning disorder NOS	Register
**Eu81z13**	[X]Learn acquisition disab NOS	Register
**Eu84112**	[X]Mental retardation with autistic features	
**Eu84200**	[X]Rett's syndrome	
**Eu84300**	[X]Other childhood disintegrative disorder	
**Eu84311**	[X]Dementia infantalis	
**Eu84312**	[X]Disintegrative psychosis	
**Eu84313**	[X]Heller's syndrome	
**Eu84400**	[X]Overactive disorder assoc mental retard/stereotype movts	
**PJ0..00**	Down's syndrome - trisomy 21	
**PJ0..11**	Mongolism	
**PJ0..12**	Trisomy 21	
**PJ0..13**	Trisomy 22	
**PJ00.00**	Trisomy 21, meiotic nondisjunction	
**PJ01.11**	Trisomy 21, mitotic nondisjunction	
**PJ02.00**	Trisomy 21, translocation	
**PJ02.11**	Partial trisomy 21 in Down's syndrome	
**PJ0z.00**	Down's syndrome NOS	
**PJ0z.11**	Trisomy 21 NOS	
**PJ1..00**	Patau's syndrome - trisomy 13	
**PJ10.00**	Trisomy 13, meiotic nondisjunction	
**PJ11.00**	Trisomy 13, mosaicism	
**PJ11.11**	Trisomy 13, mitotic nondisjunction	
**PJ12.00**	Trisomy 13, translocation	
**PJ12.11**	Partial trisomy 13 in Patau's syndrome	
**PJ1z.00**	Patau's syndrome NOS	
**PJ1z.11**	Trisomy 13 NOS	
**PJ2..00**	Edward's syndrome - trisomy 18	
**PJ20.00**	Trisomy 18, meiotic nondisjunction	
**PJ21.00**	Trisomy 18, mosaicism	
**PJ21.11**	Trisomy 18, mitotic nondisjunction	
**PJ22.00**	Trisomy 18, translocation	
**PJ22.11**	Partial trisomy 18 in Edward's syndrome	
**PJ2z.00**	Edward's syndrome NOS	
**PJ2z.11**	TRISOMY 18 NOS	
**PJ30.00**	Antimongolism syndrome	
**PJ30.11**	Deletion of long arm of chromosome 21	
**PJ31.00**	Cri-du-chat syndrome	

Read Code	Description	Used by QOF's Learning Disability*
**PJ31.11**	Deletion of short arm of chromosome 5	
**PJ32.00**	Deletion of short arm of chromosome 4	
**PJ32.11**	Wolff - Hirschorn syndrome	
**PJ33100**	Deletion of long arm of chromosome 18	
**PJ33111**	18p- syndrome	
**PJ33200**	Deletion of short arm of chromosome 18	
**PJ33211**	18q- syndrome	
**PJ33300**	Smith-Magenis syndrome	
**PJ33400**	Jacobsen syndrome	
**PJ33500**	Greig cephalopolysyndactyly syndrome	
**PJ33700**	3p deletion syndrome	
**PJ33800**	Chromosome 4q deletion syndrome	
**PJ33900**	Langer-Giedion syndrome	
**PJ33A00**	Kleefstra syndrome	
**PJ3z.00**	Monosomies and deletions from the autosomes NOS	
**PJ50.00**	Whole chromosome trisomy syndromes	
**PJ50000**	Trisomy 6	
**PJ50100**	Trisomy 7	
**PJ50200**	Trisomy 8	
**PJ50300**	Trisomy 9	
**PJ50400**	Trisomy 10	
**PJ50500**	Trisomy 11	
**PJ50600**	Trisomy 12	
**PJ50700**	Other trisomy C syndromes	
**PJ50800**	Trisomy 22	
**PJ50w00**	Whole chromosome trisomy, meitotic nondisjunction	
**PJ50x00**	Whole chromosome trisomy, mosaicism	
**PJ50x11**	Whole chromosome trisomy, mitotic nondisjunction	
**PJ50y00**	Other specified whole chromosome trisomy syndrome	
**PJ50z00**	Whole chromosome trisomy syndrome NOS	
**PJ51.00**	Partial trisomy syndromes	
**PJ51000**	Major partial trisomy	
**PJ51100**	Minor partial trisomy	
**PJ51200**	10q partial trisomy syndrome	
**PJ51300**	Trisomy 4p syndrome	
**PJ51400**	Trisomy 9p syndrome	
**PJ51500**	15q partial trisomy syndrome	
**PJ51z00**	Partial trisomy syndrome NOS	
**PJ52.00**	Trisomies of autosomes NEC	
**PJ52z00**	Trisomy of autosomes NEC NOS	
**PJ9..00**	Mowat-Wilson syndrome	
**PJyy200**	Fragile X chromosome	
**PJyy400**	Fragile X syndrome	
**PKy0.11**	Prader-Willi Syndrome	
**PKy0.12**	Prader-Willi syndrome	

Read Code	Description	Used by QOF's Learning Disability*
**PKy4.00**	William syndrome	
**PKy9300**	Prader - Willi syndrome	
**Pyu0200**	[X]Other reduction deformities of brain	
**PyuA000**	[X]Oth specif trisomies & partial trisomies of autosomes	
**R034y11**	[D]Global retardation	
**ZS34.00**	Developmental disorder of scholastic skill	
**ZS34.11**	Learning disability	

*** -** This column indicates whether the Read code was used by the Quality and Outcomes Framework (version 26 of the business rules from 2013) to identify patients on its Learning Disability register ("Register"), or used to indicate a health check ("Health Check"). The Read code **Eu818** "[X]Specific learning disability" was subsequently introduced into QOF in 2014-5 and therefore not counted in our study.

E-Table 2: Read codes used to define severe health needs

Read Code	Description	Sub-Group*
**13C5.00**	Confined to chair	Mobility severe
**13C5.11**	Chairbound	Mobility severe
**13C6.00**	Bed-ridden	Mobility severe
**13C6.11**	Bedbound	Mobility severe
**13CC.00**	Immobile	Mobility severe
**13CD.00**	Mobility very poor	Mobility severe
**13CE.00**	Mobility poor	Mobility severe
**14U5.00**	H/O: gastrostomy	PEG Feeding
**1593.00**	H/O: stress incontinence	Continence
**16F..00**	Double incontinence	Continence
**19E2.00**	Soiling - encopresis	Continence
**19E2.11**	Encopresis symptom	Continence
**19E2.12**	Soiling symptom	Continence
**19E3.00**	Incontinent of faeces	Continence
**19E3.11**	Incontinent of faeces symptom	Continence
**1A22.00**	Enuresis	Continence
**1A22000**	Nocturnal enuresis	Continence
**1A22011**	Bedwetting	Continence
**1A22100**	Daytime enuresis	Continence
**1A23.00**	Incontinence of urine	Continence
**1A24.00**	Stress incontinence	Continence
**1A24.11**	Stress incontinence - symptom	Continence
**1A26.00**	Urge incontinence of urine	Continence
**1B75.00**	Loss of vision	Severe Visual Loss
**1B77.00**	Deteriorating vision	Severe Visual Loss
**1C13.00**	Deafness	Severe Hearing impairment
**1C13300**	Bilateral deafness	Severe Hearing impairment
**1C17.00**	Hearing aid problem	Severe Hearing impairment
**2836.00**	O/E - quadriplegia	Mobility severe
**2BL..11**	O/E - deaf	Severe Hearing impairment
**2BL3.00**	O/E - significantly deaf	Severe Hearing impairment
**2BL4.00**	O/E - very deaf	Severe Hearing impairment
**2BL5.00**	O/E - completely deaf	Severe Hearing impairment
**2DG..00**	Hearing aid worn	Severe Hearing impairment
**2DH0.00**	Uses hearing loop	Severe Hearing impairment
**3930.00**	Bowels: incontinent	Continence
**3931.00**	Bowels: occasional accident	Continence
**3940.00**	Bladder: incontinent	Continence
**3941.00**	Bladder: occasional accident	Continence
**3960.00**	Dependent: chair/bed transfer	Mobility severe
**3980.00**	Immobile	Mobility severe
**3981.00**	Independent in wheelchair	Mobility severe
**3982.00**	Minimal help in wheelchair	Mobility severe
**398A.00**	Dependent on helper pushing wheelchair	Mobility severe

Read Code	Description	Sub-Group*
**6688.00**	Registered partially sighted	Severe Visual Loss
**6688.11**	Registered partially blind	Severe Visual Loss
**6689.00**	Registered blind	Severe Visual Loss
**6689.11**	Registered severely sight impaired	Severe Visual Loss
**668C.00**	Certificate of vision impairment	Severe Visual Loss
**668D.00**	Registered sight impaired	Severe Visual Loss
**7007300**	Insertion of auditory implant to brainstem	Severe Hearing impairment
**7308400**	Placement of hearing implant in external ear	Severe Hearing impairment
**7308500**	Attention to hearing implant in external ear	Severe Hearing impairment
**7308600**	Removal of hearing implant from external ear	Severe Hearing impairment
**7311A00**	Insertn bone anchors subcutaneous bone anchored hearing aid	Severe Hearing impairment
**7317C00**	Placement of hearing implant in middle ear	Severe Hearing impairment
**7317D00**	Attention to hearing implant in middle ear	Severe Hearing impairment
**7317E00**	Removal of hearing implant from middle ear	Severe Hearing impairment
**7319.00**	Attachment of bone anchored hearing prosthesis	Severe Hearing impairment
**7319000**	Insertion fixtures bone anchored hearing prosthesis Stage 1	Severe Hearing impairment
**7319100**	Insertion fixtures bone anchored hearing prosthesis Stage 2	Severe Hearing impairment
**7319200**	Reduction soft tissue for bone anchored hearing prosthesis	Severe Hearing impairment
**7319300**	Attention to fixtures for bone anchored hearing prosthesis	Severe Hearing impairment
**7319400**	One stage insert fixtures bone anchored hearing prosthesis	Severe Hearing impairment
**7319500**	Fitting external hearing prosthesis bone anchored fixtures	Severe Hearing impairment
**7319y00**	Other specified attachment bone anchored hearing prosthesis	Severe Hearing impairment
**7319z00**	Attachment of bone anchored hearing prosthesis NOS	Severe Hearing impairment
**7617.00**	Gastrostomy operations	PEG Feeding
**7617.12**	Creation of gastrostomy	PEG Feeding
**7617000**	Creation of permanent gastrostomy	PEG Feeding
**7617100**	Creation of temporary gastrostomy	PEG Feeding
**7617400**	Attention to gastrostomy tube	PEG Feeding
**7617500**	Removal of gastrostomy tube	PEG Feeding
**7617600**	Change of gastrostomy tube	PEG Feeding
**7617700**	Maintenance of percutaneous endoscopic gastrostomy tube	PEG Feeding
**7617z00**	Gastrostomy operation NOS	PEG Feeding
**7619.11**	Gastrotomy NEC	PEG Feeding
**761E300**	Temporary percutaneous endoscopic gastrostomy	PEG Feeding
**761E400**	Permanent percutaneous endoscopic gastrostomy	PEG Feeding
**761E600**	Fibreoptic endoscopic percutaneous insert gastrostomy (PEG)	PEG Feeding
**761E900**	Fibreoptic endoscopic removal of gastrostomy tube	PEG Feeding
**761EA00**	Fibreoptic endoscopic percutaneous insertion of gastrostomy	PEG Feeding
**8CJ2.00**	Percutaneous endoscopic gastrostomy feeding	PEG Feeding
**8D2..00**	Auditory aid	Severe Hearing impairment
**8D2..11**	Auditory aid provision	Severe Hearing impairment
**8D2..12**	Hearing aid provision	Severe Hearing impairment
**8D21.00**	Provide head worn hearing aid	Severe Hearing impairment
**8D22.00**	Provide body worn hearing aid	Severe Hearing impairment
**8D23.00**	Ear fitting hearing aid	Severe Hearing impairment

Read Code	Description	Sub-Group*
**8D24.00**	Replace hearing aid battery	Severe Hearing impairment
**8D25.00**	Physiolog. hearing assistance	Severe Hearing impairment
**8D2Z.00**	Auditory aid NOS	Severe Hearing impairment
**8D3..00**	Visual aid	Severe Visual Loss
**8D3..13**	Visual aid provision	Severe Visual Loss
**8D31.00**	Physiolog. visual assistance	Severe Visual Loss
**8D3Z.00**	Visual aid NOS	Severe Visual Loss
**8D73.00**	Nocturnal bladder warning syst	Continence
**8D73.11**	Enuretic alarm	Continence
**8D73.12**	Enuresis alarm	Continence
**8D9..13**	Wheel chair	Mobility severe
**8D92.00**	Self propelled wheel chair	Mobility severe
**8D93.00**	Pedal powered wheel chair	Mobility severe
**8D94.00**	Powered wheel chair	Mobility severe
**8D95.00**	Wheel chair unspecified	Mobility severe
**8D9A.00**	Attendant powered wheel chair	Mobility severe
**8D9B.00**	Wheel chair seating	Mobility severe
**8E3..00**	Deafness remedial therapy	Severe Hearing impairment
**8E3Z.00**	Deafness remedial therapy NOS	Severe Hearing impairment
**8F6..11**	Blind rehabilitation	Severe Visual Loss
**8F61.00**	Blind rehabilitation	Severe Visual Loss
**8F62.00**	Blind lead dog rehabilitation	Severe Visual Loss
**8HHC.00**	Referred for wheelchair assessment	Mobility severe
**8HlE.00**	Referral to visual impairment multidisciplinary team	Severe Visual Loss
**8M41.00**	Hearing aid requested	Severe Hearing impairment
**9m08.00**	Excluded from diabetic retinopathy screening as blind	Severe Visual Loss
**9N0b.00**	Seen in hearing aid clinic	Severe Hearing impairment
**9NfB.00**	Requires deafblind communicator guide	Severe Hearing impairment
**9NfB.00**	Requires deafblind communicator guide	Severe Visual Loss
**9NlD.00**	Seen by visual impairment teacher	Severe Visual Loss
**9R43.00**	Wheelchair in need of repair	Mobility severe
**9R44.00**	Wheelchair in good repair	Mobility severe
**9RA..00**	Wheelchair applied for	Mobility severe
**A560200**	Rubella deafness	Severe Hearing impairment
**E276.00**	Non-organic enuresis	Continence
**E276000**	Non-organic primary enuresis	Continence
**E276100**	Non-organic secondary enuresis	Continence
**E276z00**	Non-organic enuresis NOS	Continence
**E277.00**	Non-organic encopresis	Continence
**E277000**	Non-organic continuous encopresis	Continence
**E277100**	Non-organic discontinuous encopresis	Continence
**E277z00**	Non-organic encopresis NOS	Continence
**E311.00**	Severe mental retardation, IQ in range 20-34	Severe/Profound
**E312.00**	Profound mental retardation with IQ less than 20	Severe/Profound
**E312.11**	Idiocy	Severe/Profound
**Eu72.00**	[X]Severe mental retardation	Severe/Profound

Read Code	Description	Sub-Group*
**Eu72.11**	[X]Severe mental subnormality	Severe/Profound
**Eu72000**	[X]Sev mental retard with statement no or min impairm behav	Severe/Profound
**Eu72100**	[X]Sev mental retard sig impairment behav req attent/treatmt	Severe/Profound
**Eu72y00**	[X]Severe mental retardation, other impairments of behaviour	Severe/Profound
**Eu72z00**	[X]Sev mental retardation without mention impairment behav	Severe/Profound
**Eu73.00**	[X]Profound mental retardation	Severe/Profound
**Eu73.11**	[X]Profound mental subnormality	Severe/Profound
**Eu73000**	[X]Profound ment retrd wth statement no or min impairm behav	Severe/Profound
**Eu73100**	[X]Profound ment retard sig impairmnt behav req attent/treat	Severe/Profound
**Eu73y00**	[X]Profound mental retardation, other impairments of behavr	Severe/Profound
**Eu73z00**	[X]Prfnd mental retardation without mention impairment behav	Severe/Profound
**Eu81500**	[X]Severe learning disability	Severe/Profound
**Eu81700**	[X]Profound learning disability	Severe/Profound
**Eu9y000**	[X]Nonorganic enuresis	Continence
**Eu9y100**	[X]Nonorganic encopresis	Continence
**F132100**	Progressive myoclonic epilepsy	Epilepsy
**F132111**	Unverricht - Lundborg disease	Epilepsy
**F137.00**	Symptomatic torsion dystonia	Cerebral Palsy
**F137.11**	Athetoid cerebral palsy	Cerebral Palsy
**F137.12**	Athetosis - congenital	Cerebral Palsy
**F137.13**	Vogt's disease	Cerebral Palsy
**F137000**	Athetoid cerebral palsy	Cerebral Palsy
**F137011**	Vogt's disease	Cerebral Palsy
**F137100**	Double athetosis	Cerebral Palsy
**F137111**	Congenital athetosis	Cerebral Palsy
**F137y00**	Other specified symptomatic torsion dystonia	Cerebral Palsy
**F137z00**	Symptomatic torsion dystonia NOS	Cerebral Palsy
**F23..00**	Congenital cerebral palsy	Cerebral Palsy
**F23..11**	Congenital spastic cerebral palsy	Cerebral Palsy
**F23..12**	Infantile cerebral palsy	Cerebral Palsy
**F23..13**	Littles disease	Cerebral Palsy
**F23..14**	Cerebral atonia	Cerebral Palsy
**F230.00**	Congenital diplegia	Cerebral Palsy
**F230.11**	Paraplegia - congenital	Cerebral Palsy
**F230000**	Congenital paraplegia	Cerebral Palsy
**F230100**	Cerebral palsy with spastic diplegia	Cerebral Palsy
**F230z00**	Congenital diplegia NOS	Cerebral Palsy
**F231.00**	Congenital hemiplegia	Cerebral Palsy
**F232.00**	Congenital quadriplegia	Cerebral Palsy
**F232.11**	Tetraplegia - congenital	Cerebral Palsy
**F233.00**	Congenital monoplegia	Cerebral Palsy
**F233.11**	Congenital spastic foot	Cerebral Palsy
**F234.00**	Infantile hemiplegia NOS	Cerebral Palsy
**F23y.00**	Other congenital cerebral palsy	Cerebral Palsy
**F23y000**	Ataxic infantile cerebral palsy	Cerebral Palsy

Read Code	Description	Sub-Group*
**F23y100**	Flaccid infantile cerebral palsy	Cerebral Palsy
**F23y200**	Spastic cerebral palsy	Cerebral Palsy
**F23y300**	Dyskinetic cerebral palsy	Cerebral Palsy
**F23y400**	Ataxic diplegic cerebral palsy	Cerebral Palsy
**F23y500**	Worster-Drought syndrome	Cerebral Palsy
**F23y511**	Congenital suprabulbar paresis	Cerebral Palsy
**F23yz00**	Other infantile cerebral palsy NOS	Cerebral Palsy
**F23z.00**	Congenital cerebral palsy NOS	Cerebral Palsy
**F240.00**	Quadriplegia	Mobility severe
**F240.11**	Tetraplegia	Mobility severe
**F240100**	Spastic tetraplegia	Mobility severe
**F241.00**	Paraplegia	Mobility severe
**F241100**	Spastic paraplegia	Mobility severe
**F242.00**	Diplegia of upper limbs	Mobility severe
**F243.00**	Monoplegia of lower limb	Mobility severe
**F244.00**	Monoplegia of upper limb	Mobility severe
**F25..00**	Epilepsy	Epilepsy
**F250.00**	Generalised nonconvulsive epilepsy	Epilepsy
**F250200**	Epileptic seizures - atonic	Epilepsy
**F250300**	Epileptic seizures - akinetic	Epilepsy
**F250500**	Lennox-Gastaut syndrome	Epilepsy
**F250y00**	Other specified generalised nonconvulsive epilepsy	Epilepsy
**F250z00**	Generalised nonconvulsive epilepsy NOS	Epilepsy
**F251.00**	Generalised convulsive epilepsy	Epilepsy
**F251000**	Grand mal (major) epilepsy	Epilepsy
**F251011**	Tonic-clonic epilepsy	Epilepsy
**F251200**	Epileptic seizures - clonic	Epilepsy
**F251300**	Epileptic seizures - myoclonic	Epilepsy
**F251400**	Epileptic seizures - tonic	Epilepsy
**F251500**	Tonic-clonic epilepsy	Epilepsy
**F251y00**	Other specified generalised convulsive epilepsy	Epilepsy
**F251z00**	Generalised convulsive epilepsy NOS	Epilepsy
**F253.00**	Grand mal status	Epilepsy
**F253.11**	Status epilepticus	Epilepsy
**F254.00**	Partial epilepsy with impairment of consciousness	Epilepsy
**F254000**	Temporal lobe epilepsy	Epilepsy
**F254100**	Psychomotor epilepsy	Epilepsy
**F254200**	Psychosensory epilepsy	Epilepsy
**F254300**	Limbic system epilepsy	Epilepsy
**F254400**	Epileptic automatism	Epilepsy
**F254500**	Complex partial epileptic seizure	Epilepsy
**F254z00**	Partial epilepsy with impairment of consciousness NOS	Epilepsy
**F255.00**	Partial epilepsy without impairment of consciousness	Epilepsy
**F255000**	Jacksonian, focal or motor epilepsy	Epilepsy
**F255011**	Focal epilepsy	Epilepsy
**F255012**	Motor epilepsy	Epilepsy

Read Code	Description	Sub-Group*
**F255100**	Sensory induced epilepsy	Epilepsy
**F255200**	Somatosensory epilepsy	Epilepsy
**F255300**	Visceral reflex epilepsy	Epilepsy
**F255311**	Partial epilepsy with autonomic symptoms	Epilepsy
**F255400**	Visual reflex epilepsy	Epilepsy
**F255500**	Unilateral epilepsy	Epilepsy
**F255600**	Simple partial epileptic seizure	Epilepsy
**F255y00**	Partial epilepsy without impairment of consciousness OS	Epilepsy
**F255z00**	Partial epilepsy without impairment of consciousness NOS	Epilepsy
**F257.00**	Kojevnikov's epilepsy	Epilepsy
**F25B.00**	Alcohol-induced epilepsy	Epilepsy
**F25C.00**	Drug-induced epilepsy	Epilepsy
**F25D.00**	Menstrual epilepsy	Epilepsy
**F25E.00**	Stress-induced epilepsy	Epilepsy
**F25F.00**	Photosensitive epilepsy	Epilepsy
**F25X.00**	Status epilepticus, unspecified	Epilepsy
**F25y.00**	Other forms of epilepsy	Epilepsy
**F25y000**	Cursive (running) epilepsy	Epilepsy
**F25y100**	Gelastic epilepsy	Epilepsy
**F25y200**	Locl-rlt(foc)(part)idiop epilep&epilptic syn seiz locl onset	Epilepsy
**F25y300**	Complex partial status epilepticus	Epilepsy
**F25y500**	Panayiotopoulos syndrome	Epilepsy
**F25yz00**	Other forms of epilepsy NOS	Epilepsy
**F25z.00**	Epilepsy NOS	Epilepsy
**F25z.11**	Fit (in known epileptic) NOS	Epilepsy
**F2B..00**	Cerebral palsy	Cerebral Palsy
**F2B0.00**	Spastic quadriplegic cerebral palsy	Cerebral Palsy
**F2B1.00**	Spastic hemiplegic cerebral palsy	Cerebral Palsy
**F2By.00**	Other cerebral palsy	Cerebral Palsy
**F2Bz.00**	Cerebral palsy NOS	Cerebral Palsy
**F49..00**	Blindness and low vision	Severe Visual Loss
**F49..11**	Impaired vision	Severe Visual Loss
**F49..12**	Low vision	Severe Visual Loss
**F49..13**	Partial sight	Severe Visual Loss
**F49..14**	Sight impaired	Severe Visual Loss
**F490.00**	Blindness, both eyes	Severe Visual Loss
**F490000**	Unspecified blindness both eyes	Severe Visual Loss
**F490100**	Both eyes total visual impairment	Severe Visual Loss
**F490400**	Better eye: near total VI, Lesser eye: near total VI	Severe Visual Loss
**F490600**	Better eye: profound VI, Lesser eye: total VI	Severe Visual Loss
**F490900**	Acquired blindness, both eyes	Severe Visual Loss
**F490z00**	Blindness both eyes NOS	Severe Visual Loss
**F491.00**	Better eye: low vision, Lesser eye: profound VI	Severe Visual Loss
**F491000**	One eye blind, one eye low vision	Severe Visual Loss
**F491100**	Better eye: severe VI, Lesser eye: blind, unspecified	Severe Visual Loss
**F491300**	Better eye: severe VI, Lesser eye: near total VI	Severe Visual Loss

Read Code	Description	Sub-Group*
**F491400**	Better eye: severe VI, Lesser eye: profound VI	Severe Visual Loss
**F491500**	Better eye: moderate VI, Lesser eye: blind, unspecified	Severe Visual Loss
**F491700**	Better eye: moderate VI, Lesser eye: near total VI	Severe Visual Loss
**F491z00**	One eye blind, one eye low vision NOS	Severe Visual Loss
**F492.00**	Low vision, both eyes	Severe Visual Loss
**F492000**	Low vision, both eyes unspecified	Severe Visual Loss
**F492200**	Better eye: severe VI, Lesser eye: severe VI	Severe Visual Loss
**F492300**	Better eye: moderate VI, Lesser eye: low vision unspecified	Severe Visual Loss
**F492400**	Better eye: moderate VI, Lesser eye: severe VI	Severe Visual Loss
**F492500**	Better eye: moderate VI, Lesser eye: moderate VI	Severe Visual Loss
**F492z00**	Low vision, both eyes NOS	Severe Visual Loss
**F493.00**	Visual loss, both eyes unqualified	Severe Visual Loss
**F494.00**	Legal blindness USA	Severe Visual Loss
**F497.00**	Severe visual impairment, binocular	Severe Visual Loss
**F498.00**	Moderate visual impairment, binocular	Severe Visual Loss
**F49z.00**	Visual loss NOS	Severe Visual Loss
**F49z.11**	Acquired blindness	Severe Visual Loss
**F4H7300**	Cortical blindness	Severe Visual Loss
**F581211**	Noise induced deafness	Severe Hearing impairment
**F59..11**	Deafness	Severe Hearing impairment
**F590.11**	Conductive deafness	Severe Hearing impairment
**F591.13**	Perceptive deafness	Severe Hearing impairment
**F591211**	Nerve deafness	Severe Hearing impairment
**F591400**	Congenital sensorineural deafness	Severe Hearing impairment
**F591500**	Ototoxicity - deafness	Severe Hearing impairment
**F591511**	Drug ototoxicity - deafness	Severe Hearing impairment
**F591800**	Congenital prelingual deafness	Severe Hearing impairment
**F592.00**	Mixed conductive and sensorineural deafness	Severe Hearing impairment
**F593.00**	Deaf mutism, NEC	Severe Hearing impairment
**F594.00**	High frequency deafness	Severe Hearing impairment
**F595.00**	Low frequency deafness	Severe Hearing impairment
**F596.00**	Maternally inherited deafness	Severe Hearing impairment
**F598.00**	Moderate acquired hearing loss	Severe Hearing impairment
**F599.00**	Severe acquired hearing loss	Severe Hearing impairment
**F59A.00**	Profound acquired hearing loss	Severe Hearing impairment
**F59A.11**	Deafened	Severe Hearing impairment
**F59z.00**	Deafness NOS	Severe Hearing impairment
**F59z.11**	Chronic deafness	Severe Hearing impairment
**Fyu9.00**	[X]Cerebral palsy and other paralytic syndromes	Cerebral Palsy
**Fyu9000**	[X]Other infantile cerebral palsy	Cerebral Palsy
**Fyu9100**	[X]Other specified paralytic syndromes	Cerebral Palsy
**FyuU000**	[X]Deaf mutism, not elsewhere classified	Severe Hearing impairment
**K198.00**	Stress incontinence	Continence
**K586.00**	Stress incontinence - female	Continence
**Kyu5A00**	[X]Other specified urinary incontinence	Continence
**P40z.11**	Deafness due to congenital anomaly NEC	Severe Hearing impairment

Read Code	Description	Sub-Group*
**R00A.00**	[D] Poor mobility	Mobility severe
**R00C.00**	[D]Immobility	Mobility severe
**R076.00**	[D]Incontinence of faeces	Continence
**R076000**	[D]Encopresis NOS	Continence
**R076100**	[D]Sphincter ani incontinence	Continence
**R076z00**	[D]Incontinence of faeces NOS	Continence
**R083.00**	[D]Incontinence of urine	Continence
**R083000**	[D]Enuresis NOS	Continence
**R083100**	[D]Urethral sphincter incontinence	Continence
**R083200**	[D] Urge incontinence	Continence
**R083z00**	[D]Incontinence of urine NOS	Continence
**SJ15.12**	Deafness - traumatic - NOS	Severe Hearing impairment
**Z1J..00**	Procedures to aid continence	Continence
**Z6R3.00**	Wheelchair dancing therapy	Mobility severe
**Z6R8100**	Wheelchair sport	Mobility severe
**Z6X1.00**	Wheelchair transfer practice	Mobility severe
**Z6Z..00**	Wheelchair education	Mobility severe
**Z6Z1.00**	Wheelchair use training	Mobility severe
**Z6Z1200**	Propelling wheelchair training	Mobility severe
**Z6Z1300**	Controlling electric wheelchair training	Mobility severe
**Z8B5.00**	Ability to use hearing aid	Severe Hearing impairment
**Z8B5100**	Able to use hearing aid	Severe Hearing impairment
**Z8B5200**	Unable to use hearing aid	Severe Hearing impairment
**Z8B5300**	Does use hearing aid	Severe Hearing impairment
**Z8B5311**	Uses hearing aid	Severe Hearing impairment
**Z8B5400**	Does not use hearing aid	Severe Hearing impairment
**Z8B5500**	Difficulty using hearing aid	Severe Hearing impairment
**Z911.00**	Hearing aid procedure	Severe Hearing impairment
**Z911100**	Fit hearing aid	Severe Hearing impairment
**Z911300**	Adjust hearing aid settings	Severe Hearing impairment
**Z911400**	Changing hearing aid battery	Severe Hearing impairment
**Z911500**	Checking hearing aid	Severe Hearing impairment
**Z911700**	Switching on hearing aid	Severe Hearing impairment
**Z911800**	Turning off hearing aid	Severe Hearing impairment
**Z911900**	Putting on hearing aid	Severe Hearing impairment
**Z911A00**	Listening for feedback whistle of hearing aid	Severe Hearing impairment
**Z911B00**	Attention to hearing aid	Severe Hearing impairment
**Z911E00**	Fit ear mould for existing hearing aid	Severe Hearing impairment
**Z96..00**	Provision for visual and hearing impairment	Severe Visual Loss
**Z961.00**	Provision of guide help for visual and hearing impairment	Severe Visual Loss
**Z9E2.00**	Optical low vision aid provision	Severe Visual Loss
**Z9E3.00**	Provision of optical low vision aid - near	Severe Visual Loss
**Z9E3100**	Provision of magnifier low vision aid - near	Severe Visual Loss
**Z9E3200**	Provision of low vision hand magnifier	Severe Visual Loss
**Z9E3300**	Provision of low vision stand magnifier	Severe Visual Loss
**Z9E3500**	Provision of spectacle low vision aid - near	Severe Visual Loss

Read Code	Description	Sub-Group*
**Z9E3600**	Provision of telescopic spectacles	Severe Visual Loss
**Z9E3700**	Provision of spectacle magnifier	Severe Visual Loss
**Z9E3900**	Near low vision aid - clip-on spectacle magnifier	Severe Visual Loss
**Z9E3A00**	Provision of spectacle telescope	Severe Visual Loss
**Z9E3B00**	Near low vision aid - integral spectacle telescope	Severe Visual Loss
**Z9E3C00**	Near low vision aid - clip-on spectacle telescope	Severe Visual Loss
**Z9E3D00**	Near low vision aid - extra cap for telescope	Severe Visual Loss
**Z9E3E00**	Provision of headband telescope	Severe Visual Loss
**Z9E4.00**	Provision of optical low vision aid - distance	Severe Visual Loss
**Z9E5.00**	Provision of non-optical low vision aid	Severe Visual Loss
**Z9E5200**	Provision of closed circuit television	Severe Visual Loss
**Z9E5300**	Provision of image intensifier	Severe Visual Loss
**Z9E5400**	Provision of ancillary low vision aid	Severe Visual Loss
**Z9E5700**	Provision of work board	Severe Visual Loss
**Z9E6.00**	Provision of visual appliance	Severe Visual Loss
**Z9E6500**	Provision of audiotaped services	Severe Visual Loss
**Z9E6600**	Provision of talking book	Severe Visual Loss
**Z9E8100**	Hearing aid provision	Severe Hearing impairment
**Z9E8111**	Auditory aid provision	Severe Hearing impairment
**Z9EA.00**	Provision of incontinence appliance	Continence
**Z9EA100**	Provision of nocturnal bladder warning system	Continence
**Z9EA111**	Provision of enuresis alarm	Continence
**Z9EA112**	Provision of enuretic alarm	Continence
**Z9EH400**	Provision of wheelchair	Mobility severe
**Z9MO.00**	Enuresis support	Continence
**ZC65200**	Gastrostomy feeding	PEG Feeding
**ZC65300**	Percutaneous endoscopic gastrostomy feeding	PEG Feeding
**ZC65311**	PEG - Percutaneous endoscopic gastrostomy feeding	PEG Feeding
**ZC65400**	Button gastrostomy feeding	PEG Feeding
**ZC65500**	Jejunostomy feeding	PEG Feeding
**ZE83200**	Hearing for loud voice impaired	Severe Hearing impairment
**ZE84200**	Hearing for voice impaired	Severe Hearing impairment
**ZE87.00**	Hearing loss	Severe Hearing impairment
**ZE87.11**	Deafness	Severe Hearing impairment
**ZE87.13**	Hard of hearing	Severe Hearing impairment
**ZE87.16**	HL - Hearing loss	Severe Hearing impairment
**ZE87.17**	HOH - Hard of hearing	Severe Hearing impairment
**ZL22400**	Under care of continence nurse	Continence
**ZN56800**	Blind telephone user	Severe Visual Loss
**ZN56900**	Deaf telephone user	Severe Hearing impairment
**ZO2..00**	Unable to mobilise	Mobility severe
**ZO4..00**	Does not mobilise	Mobility severe
**ZO72.00**	Unable to mobilise indoors	Mobility severe
**ZO74.00**	Does not mobilise indoors	Mobility severe
**ZO75.00**	Difficulty mobilising indoors	Mobility severe
**ZO92.00**	Unable to mobilise using mobility aids	Mobility severe

Read Code	Description	Sub-Group*
**ZO93.00**	Does mobilise using aids	Mobility severe
**ZO94.00**	Does not mobilise using mobility aids	Mobility severe
**ZO96.00**	Ability to mobilise using wheelchair	Mobility severe
**ZO96.11**	Wheelchair mobility	Mobility severe
**ZO96100**	Able to mobilise using wheelchair	Mobility severe
**ZO96200**	Unable to mobilise using wheelchair	Mobility severe
**ZO96300**	Does mobilise using wheelchair	Mobility severe
**ZO96311**	Mobilises using wheelchair	Mobility severe
**ZO96400**	Does not mobilise using wheelchair	Mobility severe
**ZO96500**	Difficulty mobilising using wheelchair	Mobility severe
**ZOC6200**	Unable to get in and out of a chair	Mobility severe
**ZOC6400**	Does not get in and out of a chair	Mobility severe
**ZOC8200**	Unable to get out of a chair	Mobility severe
**ZOC8400**	Does not get out of a chair	Mobility severe
**ZOC9200**	Unable to get on and off a bed	Mobility severe
**ZOC9400**	Does not get on and off a bed	Mobility severe
**ZOCA200**	Unable to get on a bed	Mobility severe
**ZOCB200**	Unable to get off a bed	Mobility severe
**ZOCB400**	Does not get off a bed	Mobility severe
**ZOD2.00**	Unable to move in bed	Mobility severe
**ZOD4.00**	Does not move in bed	Mobility severe
**ZOD6200**	Unable to roll over in bed	Mobility severe
**ZOD6211**	Unable to turn over in bed	Mobility severe
**ZOD7500**	Difficulty turning onto side in bed	Mobility severe
**ZOD8200**	Unable to move up and down bed	Mobility severe
**ZT12711**	Voice associated with hearing loss	Severe Hearing impairment
**ZV44100**	[V]Has gastrostomy	PEG Feeding
**ZV45G00**	[V]Presence of external hearing-aid	Severe Hearing impairment
**ZV45N00**	[V]Bone anchored hearing aid in situ	Severe Hearing impairment
**ZV46200**	[V]Dependence on wheelchair	Mobility severe
**ZV4L011**	[V] Poor mobility	Mobility severe
**ZV53200**	[V]Fitting or adjustment of hearing aid	Severe Hearing impairment
**ZV53800**	[V]Fitting or adjustment of wheelchair	Mobility severe
**ZV53D00**	[V]Adjustment and management of implanted hearing device	Severe Hearing impairment
**ZV55100**	[V]Attention to gastrostomy	PEG Feeding

* - To be classed as having severe health needs, must be “Severe/Profound” or have 2 or more of the following in addition to an ID diagnosis: epilepsy, cerebral palsy or significant mobility problem, severe visual impairment, severe hearing impairment, a continence problem or use of PEG feeding.

E-Table 3: Read codes used to identify communal or shared living accommodation

Read Code	Description
**13F4.00**	Warden attended
**13F4.11**	Lives in warden controlled accommodation
**13F4000**	Resident in sheltered accommodation
**13F5.00**	Part III accommodation
**13F5.11**	Part 3 accommodation
**13F5100**	Part III accommodation arranged
**13F5111**	Part 3 accommodation arranged
**13F5200**	Resident in part III accommodation
**13F6.00**	Nursing/other home
**13F6100**	Lives in a nursing home
**13F7.00**	Residential institution
**13F7100**	Lives in a welfare home
**13F7200**	Lives in an old peoples home
**13F7300**	Lives in a childrens home
**13F7400**	Admitted to a children's home
**13F8100**	Long stay hospital inpatient
**13F9.00**	Living in hostel
**13F9.11**	Living in sheltered accomodatn
**13FK.00**	Lives in a residential home
**13FS.00**	Long stay hospital inpatient
**13FT.00**	Lives in an old peoples home
**13FV.00**	Lives in a welfare home
**13FX.00**	Lives in care home
**13FY.00**	Lives in a children's unit
**Z177100**	24 hour care
**Z177500**	Custodial care
**Z177C00**	Residential care
**Z177D00**	Local authority residential care
**Z177D11**	LA - local authority residential care
**ZU37.00**	Lives in a community
**ZU37100**	Lives in a school community
**ZU37200**	Lives in boarding school
**ZV60600**	[V]Institution resident
**ZV60611**	[V]Boarding school resident
**ZV60700**	[V]Sheltered housing
**ZU37100**	Lives in a school community

E-Table 4: Ambulatory care sensitive conditions (ACSCs) and ICD-10 codes used to define them

Conditions	ICD-10 Code
**Angina**	I20, I24.0, I24.8-I24.9
**Aspiration**	J69.0, J69.8
**Asthma**	J45-J46
**Cellulitis**	L03-L04, L08, L88, L98.0, L98.3
**Congestive heart failure**	I11.0, I50, J81
**Constipation**	K59.0
**Convulsions/epilepsy**	G40-G41, R56, O15
**Chronic obstructive pulmonary disease (COPD)**	J41-J44, J47
**Dehydration & gastroenteritis**	E86, K52.2, K52.8, K52.9
**Dental conditions**	A69.0, K02-K06, K08, K09.8, K09.9, K12-K13
**Diabetes complications**	E10.0-E10.8, E11.0-E11.8, E12.0-E12.8, E13.0-E13.8, E14.0-E14.8
**Ear, nose and throat infections**	H66-H67, J02-J03, J06, J31.2
**Gangrene**	R02
**Gastro-oesophageal reflux disease**	K21
**Hypertension**	I10, I11.9
**Iron deficiency anaemia**	D50.1, D50.8-D50.9
**Influenza**	J10-J11
**Nutritional deficiencies**	E40-E43, E55, E64.3
**Pelvic inflammatory disease**	N70, N73-N74
**Perforated/bleeding ulcers**	K25.0-K25.2, K25.4-K25.6, K26.0-K26.2, K26.4-K26.6, K27.0-K27.2, K27.4-K27.6, K28.0-K28.2, K28.4-K28.6
**Pneumonia & other acute lower respiratory tract infection (LRTI)**	J13-J14, J15.3-J15.4, J15.7, J15.9, J16.8, J18.1, J18.8, J20-J20.2, J20.8, J20.9, J22
**Tuberculosis & other vaccine preventable**	A15-A16, A19, A35-A37, A80, B05-B06, B16.1, B16.9, B18.0-B18.1, B26, G00.0, M01.4
**Urinary tract infection (UTI)/pyelonephritis**	N10-N12, N13.6, N39.0
